# Patient-specific fine-tuning of convolutional neural networks for follow-up lesion quantification

**DOI:** 10.1117/1.JMI.7.6.064003

**Published:** 2020-12-17

**Authors:** Mariëlle J. A. Jansen, Hugo J. Kuijf, Ashis K. Dhara, Nick A. Weaver, Geert Jan Biessels, Robin Strand, Josien P. W. Pluim

**Affiliations:** a University Medical Center Utrecht and Utrecht University, Image Sciences Institute, Utrecht, The Netherlands; b Uppsala University, Center for Image Analysis, Department of Information Technology, Uppsala, Sweden; c University Medical Center Utrecht, Brain Center Rudolf Magnus, Department of Neurology, Utrecht, The Netherlands

**Keywords:** convolutional neural network, magnetic resonance imaging, patient-specific

## Abstract

**Purpose:** Convolutional neural network (CNN) methods have been proposed to quantify lesions in medical imaging. Commonly, more than one imaging examination is available for a patient, but the serial information in these images often remains unused. CNN-based methods have the potential to extract valuable information from previously acquired imaging to better quantify lesions on current imaging of the same patient.

**Approach:** A pretrained CNN can be updated with a patient’s previously acquired imaging: patient-specific fine-tuning (FT). In this work, we studied the improvement in performance of lesion quantification methods on magnetic resonance images after FT compared to a pretrained base CNN. We applied the method to two different approaches: the detection of liver metastases and the segmentation of brain white matter hyperintensities (WMH).

**Results:** The patient-specific fine-tuned CNN has a better performance than the base CNN. For the liver metastases, the median true positive rate increases from 0.67 to 0.85. For the WMH segmentation, the mean Dice similarity coefficient increases from 0.82 to 0.87.

**Conclusions:** We showed that patient-specific FT has the potential to improve the lesion quantification performance of general CNNs by exploiting a patient’s previously acquired imaging.

## 1 Introduction

Lesion quantification is an important step in medical image analysis. Over the past few years, convolutional neural network (CNN)-based lesion quantification methods have been proposed for use in medical image analysis^1^^–^^5^ to aid radiologists in the detection and segmentation of lesions. For some diseases or treatments, there is a clinical need to perform multiple scans over time, for example, to monitor disease progression or treatment response. For these patients, more than one imaging examination is available. The previously acquired scan, i.e., the baseline scan, contains patient-specific information that could be exploited by the CNN-based method to better quantify the current scan of the same patient, i.e., the follow-up scan.

The conventional way to train a CNN is to provide a (large) example dataset in which the lesion of interest has been identified or segmented. The trained CNN can then be applied in comparable data sets. However, CNN-based methods do not always provide a satisfying performance for clinical practice. Differences in image quality and variations among patients are often not fully covered within the method and can cause unsatisfactory results. As a result of training a CNN on a general population, the CNN is not fully adapted to the specific details of an individual patient.

Updating a CNN toward the specific features of a patient could improve the performance of the CNN for that patient.^6^^,^^7^ A previously acquired scan of the same patient can be used for this purpose since it is already available and can serve as a reference during examination of the follow-up scan. We, therefore, propose a method to enhance the performance of lesion quantification in a follow-up scan with a patient-specific fine-tuning (FT) approach. To illustrate our envisioned workflow: at first visit, a patient’s medical images are processed by the base CNN and the results are visually inspected and corrected where needed. At a follow-up visit, the base CNN can be updated with the corrected results from the first visit of the same patient to improve the performance of the method on the new scan.

FT a pretrained CNN has the benefit of obtaining good results using only a small data set during the FT step, and has successfully been applied in previous studies. For example, medical image domain knowledge has been transferred to a CNN pretrained on natural images by FT the CNN with medical images.^8^^,^^9^ Pretrained CNNs have been fine-tuned toward the features of one specific image, resulting in better segmentations of that image.^10^^,^^11^

A patient-specific fine-tuned CNN has learned the specific features of abnormalities and the surrounding healthy tissue of a patient to improve the detection or segmentation in a follow-up scan. This approach is based on the assumption that the baseline and follow-up scan of a patient share the features of healthy tissue and abnormalities.

We present and evaluate the patient-specific FT approach on two applications; the detection of liver metastases on magnetic resonance imaging (MRI) and the segmentation of brain white matter hyperintensities (WMH) on MRI.

The detection of (new) liver metastases is important to monitor disease progression and for treatment planning. Treatment selection is based on the detection findings, i.e., the location and the number of metastases. Disease progression is monitored by follow-up scans. Detection of these metastases is the primary goal.

WMH is a common radiological finding in the elderly population, and is generally considered to reflect cerebral small vessel disease.^12^ The presence and extent of WMH are associated with cognitive decline and dementia.^12^^–^^14^ In particular, progression of WMH over time has been linked to cognitive decline and risk of dementia.^15^^,^^16^ Quantification of WMH volume changes over time could contribute to the monitoring of disease progression and provide clinicians with relevant prognostic information. WMH segmentation for the purpose of volume measurements is the primary goal.

Furthermore, different aspects of patient-specific FT are studied using the two applications.

## 2 Materials and Methods

**Fig. 1:**
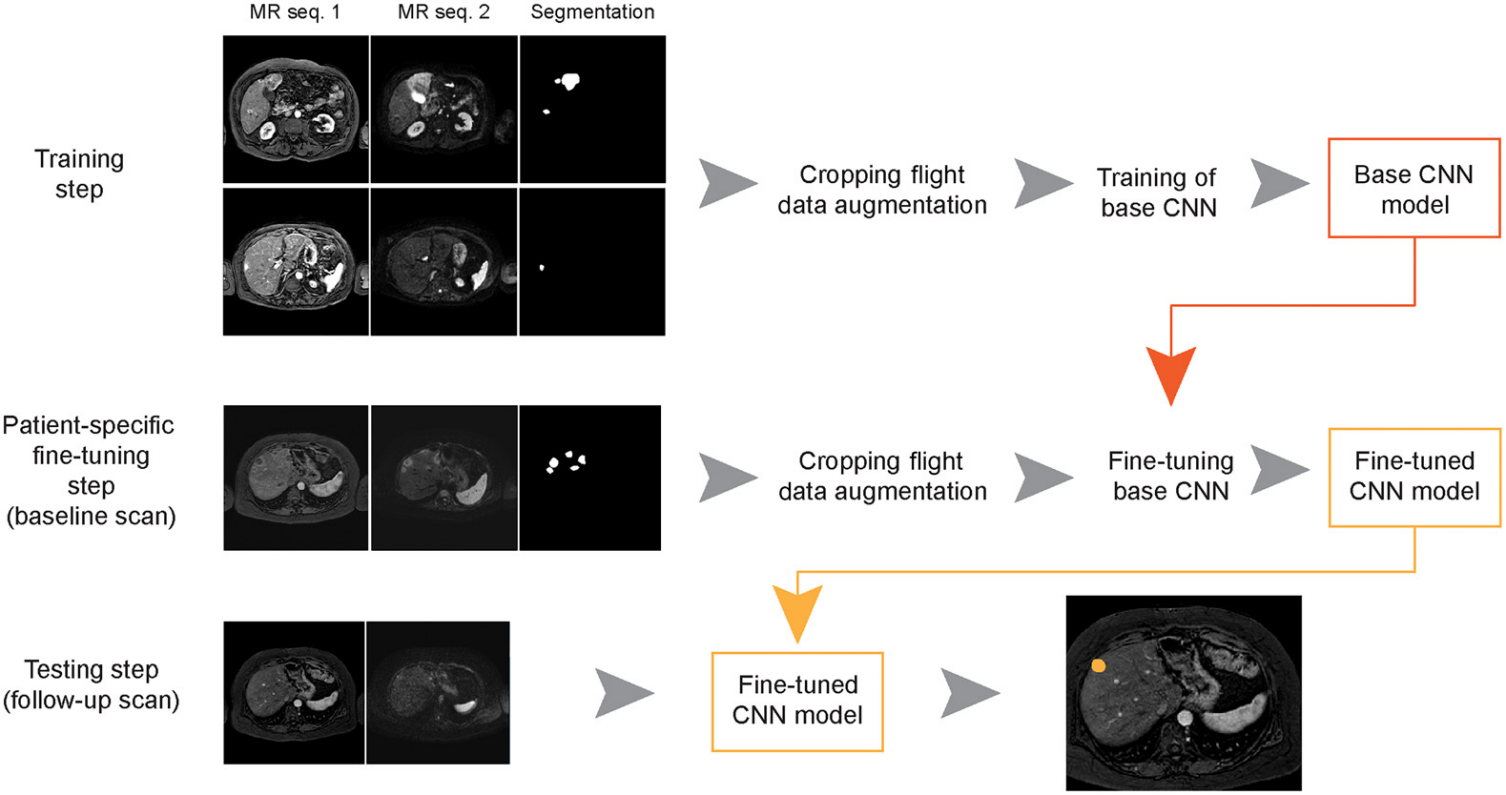
Proposed lesion quantification framework, shown with the liver MRI as an example. First a base CNN is trained with a training set consisting of multiple patients. Next, the base CNN is refined in the patient-specific FT step using a previous MRI exam of a patient (the baseline scan). The fine-tuned CNN is used to detect or segment lesions in a follow-up MRI scan of the same patient. The images are cropped to focus of the organ of interest. The cropped image size is 128×128  pixels.

The proposed lesion quantification framework is shown in Fig. 1. First, a CNN is trained using the training set, referred to as the base CNN model. Next, this base CNN is refined for each individual patient in the patient-specific FT step. A previous MRI scan of a patient, referred to as the baseline scan, is used to fine-tune the network to the specific features of that patient and its lesions. During the testing step, this patient-specific CNN model is used to detect or segment lesions in a follow-up MRI scan of the same patient. Two different MRI sequences are used to train and test the CNN model.

### 2.1 Data

The patient-specific FT approach is demonstrated on two different data sets: abdominal MRI for liver metastasis detection and brain MRI for WMH segmentation. The University Medical Center Utrecht (UMCU) Medical Ethical Committee has reviewed and approved this study and has waived informed consent due to the study’s retrospective nature.

#### 2.1.1 Liver metastases detection

In this study, abdominal MRI of 47 patients with liver metastases from the UMCU, the Netherlands, were included. Around 16 of them had at least two consecutive MRI examinations within three months to a year. All patients underwent a clinical MRI examination, including dynamic contrast-enhanced (DCE) MRI series and diffusion weighted (DW) MRI, acquired on a 1.5T scanner (Philips, Best, the Netherlands). These two MRI sequences were used to train the CNN.

The DCE-MRI series was acquired in six breath holds with one to five three-dimensional (3D) images per breath hold, with a total of 16 3D images. The DW-MRI was acquired with three b-values: 10, 150, and $1000\;{s/\mathrm{mm}}^{2}$. The DCE-MRI was corrected for motion using a principle component analysis-based groupwise registration.^17^ Intensity normalization was applied to both MRI sequences and the DW-MRI was registered to the DCE-MRI by a rigid transformation, followed by a b-spline transformation (the parameter files used are available at elastix.bigr.nl/wiki/index.php/Par0057). The resulting images have 100 slices and matrix sizes of 256×256. The oxel size is $1.543\times 1.543\times 2\;{\mathrm{mm}}^{3}$.

The liver metastases were manually segmented on the DCE-MRI by a radiologist in training and verified by a radiologist with more than ten years of experience. The data set included mainly colorectal metastases, neuroendocrine metastases, and some other metastasis types (i.e., other gastrointestinal metastases and breast metastases). On average, 30% of the liver slices contained liver metastases. Liver masks were automatically obtained using our previously developed segmentation method.^18^

MRI data from 31 patients of which only one MRI examination was available were used to train the base CNN. The slices for training were limited to only the slices containing liver metastases for a more balanced data set. Slices without liver metastases were not included in the training set. This resulted in a total number of 798 two-dimensional (2D) slices; 85% of the slices were used for training and 15% for validation. The remaining 16 patients, with available baseline and follow-up scans, were used for testing. The scans had an average of six metastases per patient, ranging from 1 to 31 metastases.

#### 2.1.2 WMH segmentation

The brain MRI data of 80 memory clinic patients with WMH were included in the study. Around 20 of the patients were from the Dutch Parelsnoer Institute—neurodegenerative diseases study,^19^ from the UMC Utrecht, the Netherlands, and the remaining 60 were from the WMH challenge training set.^20^ The 20 patients from UMC Utrecht had two MRI examinations, with two years between the two examinations.

All MRI exams were acquired with a similar protocol, with a T2-weighted-fluid-attenuated inversion recovery (FLAIR) MRI and a T1-weighted MRI, acquired on a 3T scanner.^21^ These two MRI sequences were used to train the CNN. Both MRI sequences were bias field corrected, and the T1-weighted MRI were registered to the FLAIR images; more details can be found in Kuijf et al.^20^ All images were resized to a matrix size of 240×240 and 48 axial slices. Voxel size was $0.958\times 0.958\times 3.00\;{\mathrm{mm}}^{3}$.

The WMH were manually segmented on the axial slices of FLAIR images by an experienced researcher in accordance with the STRIVE criteria.^13^ WMH segmentation was performed with in-house developed software on MeVisLab (MeVis Medical Solutions AG, Bremen, Germany). On average, 63% of the brain slices contained WMH. Brain masks were obtained using the SPM software.^22^

The MRI data of 60 patients with a single MRI examination from the WMH challenge were used for training. The slices for training were limited to only the slices with WMH lesions present for a more balanced data set. Slices without WMH lesions were not included in the training set. This resulted in a total of 1383 2D slices; about 85% of the slices were used for training and 15% for validation. The remaining 20 patients, with a baseline and follow-up scan, were used for testing. These scans had an average of 65 WMH per patient (range 21 to 117).

### 2.2 Base CNN Model

**Fig. 2:**
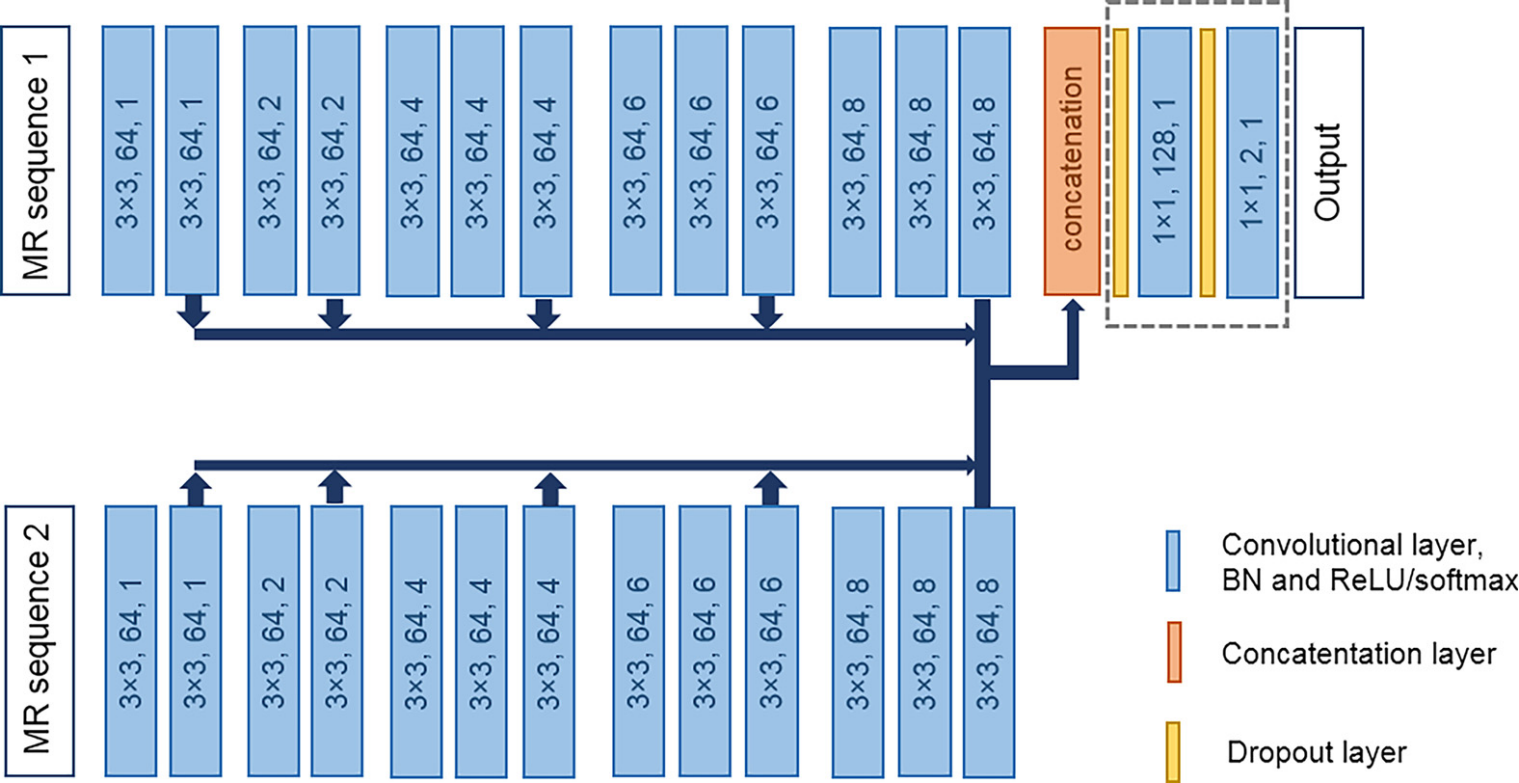
Fully convolutional network architecture for lesion quantification. The blue blocks represent the convolutional layers, BN, and ReLU, or Softmax activation. The size of the kernel of each convolutional layer is given in the block, followed by the number of kernels and the dilation rate of the kernel. The dashed line indicates the trainable layers during the patient-specific FT step.

The overall architecture of the CNN was inspired by our earlier work on liver metastasis detection.^18^ This fully convolutional architecture includes elements from the P-net architecture, which has proven to be efficient for FT.^10^ As most MRI exams usually consist of multiple MRI sequences, we modified the original P-net to include a dual pathway that can process two MRI sequences, each in a separate pathway to extract specific feature maps for those sequences. The input image for each pathway was one MRI sequence. If the MRI sequence had multiple instances, such as the phases of the abdominal DCE-MRI, the 2D images were combined into one input image with the instances as channels. Variations on the network architecture have been tested in our earlier work.^18^ From Fig. 2, we can see an overview of the fully convolutional network architecture.

Each pathway had 13 convolutional layers (two convolutional layers and 11 dilated convolutional layers^23^ with varying dilation rates), each having 3×3 convolution and 64 kernels, split into five blocks. The feature maps at the end of each block of each pathway were concatenated in the third dimension, resulting in a feature map with 640 kernels, and were passed to two convolutional layers with a 1×1 convolution with 128 and two kernels, respectively. This resulted in a receptive field of 123×123  pixels.

During training, categorical cross-entropy was used as the loss function. This loss function was calculated on a pixel-level resulting in detection by segmentation. Rectified linear unit (ReLU) activation and batch normalization (BN) were used in all the convolutional layers, except for the final layer, which had a Softmax activation. Two dropout layers were applied before and after the second to last layer. The dropout rate was set to 0.2. The classes were weighted based on class frequencies using a weighted loss function. The class weights were set to 1 for the background class and to 5 for the lesion class. He uniform was used as initializer and Adam as optimizer with a learning rate of 0.0001. The network was trained for 10,000 iterations, with four images per minibatch. The network with the lowest validation loss was used as the final network.

Around 25 patches of 128×128  pixels were taken from each slice for data augmentation. The patches originated from the organ of interest and have overlapping areas. Online data augmentation was applied by random rotation of the patches, with rotation angles of ±45  deg.

### 2.3 Patient-Specific Fine-Tuning

In the patient-specific FT step, the last two layers of the base CNN were further trained with the baseline scan of a patient to improve the lesion quantification results in the follow-up scan of the same patient. Only the last two layers were updated, as these combine the feature maps of the previous blocks of the two pathways and to ensure a fast FT step.

The CNN was refined by continuing training of the base CNN with the batch norm and the weights of all layers frozen, i.e., untrainable, except for those of the last two layers. The last two layers combine the feature maps of the previous layers to classify each pixel. FT of only these two layers decreases the computational complexity while effectively improving the CNN model. The two trainable layers are indicated with the dashed line box in Fig. 2.

The same loss function, optimizer, and learning rate were used as the ones during the training of the base CNN. The CNN was refined with four images per minibatch and the number of iterations was optimized during the experiments, see the experiments section. The images used for FT were MRI slices of the patient’s baseline scan. The baseline scan was annotated earlier by an expert and only slices with at least one lesion present were included in the FT step. Five patches of 128×128  pixels were taken within the organ of interest region from each slice, four patches from the corners of the region and one center patch, removing air and tissues not of interest from the input images. The patches were randomly rotated, with rotation angles of ±45  deg. Rotation of the patches included the variance in the positioning of the body during the MRI examination.

### 2.4 Lesion Quantification on Follow-Up Scan

The follow-up data were processed by the individual patient-specific CNN, i.e., the base CNN fine-tuned with the baseline scan of that patient. The probability output of the network was masked by the liver or brain mask.

For the evaluation, the masked probability output was postprocessed to a binary image. A threshold of 0.5 was applied to the Softmax output of the network and morphological closing with a structuring element of 3×3×3 was applied to fill holes. For the liver data set, the morphological closing was followed by a morphological opening with a plus-shaped structuring element of 3×3 to remove noise pixels. This was not applied to the brain data set, because WMHs of 1 pixel occur frequently. For the liver data set, the resulting binary image was divided into separate objects representing individual lesions, using voxel clustering with 26-neighborhood connection.

## 3 Experiments

The set up for the base CNN model and the process of FT the CNN, as explained above, are similar for both the liver metastases detection and the WMH segmentation. This means that the detection is done pixelwise instead of on an object-level during training and FT. The same experiments are conducted. Only the evaluation is specified to the task that is most relevant in clinical routine: liver metastases detection and WMH segmentation.

### 3.1 Liver Metastases Detection

The liver metastases detection efficacy was evaluated on object level using the true positive rate (TPR), the number of false positives per case (FPC), and the F1 score. As the values of the TPR, FPC, and F1 score do not have a normal distribution, the median and interquartile range (IQR) were reported and the Wilcoxon signed-rank test was used to test for significant differences.

The TPR was calculated as the number of true positive objects divided by the total number of true lesion objects. The true lesion objects were true positive objects (detected lesions) and false negative objects (missed lesions). A lesion was considered detected, and thus a true positive object, when the manual annotation and the predicted segmentations had an overlap >0. The FPC was calculated as the number of detected objects not overlapping with any true lesion object. The F1 score was calculated as $\frac{2*\text{recall}*\text{precision}}{\text{recall}+\text{precision}}$, where recall was the TPR as defined above and precision was the number of true positive objects divided by the total number of detected objects (both true positive and false positive objects).

### 3.2 WMH Segmentation

WMH segmentation on brain MRIs was evaluated per MRI exam using the overall Dice similarity coefficient (DSC) and the absolute volume difference (AVD). The Dice score was calculated as $\frac{2*X\cap Y}{X+Y}$, where X is the automatic segmentation and Y is the manual annotation. The AVD was calculated as $\frac{\mathrm{abs}[V(X)-V(Y)]}{V(Y)}*100\%$, where V(X) is the automatic segmentation volume and V(Y) the manual annotation volume. The mean and standard deviation (SD) are reported and the paired Student’s t-test is used to test for significant differences.

The following experiments analyzed different elements of patient-specific FT; the number of iterations, the number of slices presented during FT, and a weighting scheme.

### 3.3 Number of Iterations

The duration of the FT should be adapted to the similarity between the baseline and the follow-up scan, as the baseline scan can have a different appearance to the follow-up scan. For example, liver metastases show changes in shape and size after treatment, and WMH is known to progress from punctuate to confluent lesions over time. If the CNN is fine-tuned for too few iterations, the CNN will not be patient specific enough. If the number of iterations is too high, the risk of overfitting toward the baseline scan increases. The number of iterations was studied to provide the optimal number of iterations for general use of patient-specific FT. This optimal number of iterations will be used in further experiments. The number of iterations explored ranges from 50 to 1000.

### 3.4 Number of Slices

The possibility of FT with only one or two slices—instead of an entire scan—is studied to investigate the option of limiting human efforts. For the dataset used in the current study, the full manual annotation was available, but this is not common in clinical routine. Annotating or correcting the annotation of the baseline scan on one or two slices would be a smaller effort than annotating a full image in case a good annotation does not yet exist. In these experiments, the number of slices that is offered to the CNN is one, two, or all slices with lesions or the full organ of interest.

Two options were explored for the slice selection procedure. The first includes all slices for slice selection and the second option only includes slices with lesions present for slice selection. The slice selection is based on the Softmax probability outcome of the base CNN on the baseline scan. The slices with an average Softmax probability closest to 0.5 are selected, assuming that these slices contain the most valuable information for FT the CNN.

### 3.5 Weighting

Weighting false negatives, false positives, and true positives, obtained after performing the lesion quantification with the base CNN on the baseline scan, will redefine what we wanted the CNN to learn during FT. The weights depended on which task was performed.

For the detection task, the objective was to detect the metastases on liver MRI. The weights of the missed metastases are set to the highest value (five). The weights of the true positive pixels in detected metastases were set to two, with the weights of false negatives and the false positive pixels connected to the detected metastases set to 0. The background and false positive objects weights were set to 1. In this manner, the fine-tuned CNN would be more inclined to label pixels as liver metastasis. Over- or under-segmentation of detected metastases was not considered incorrect labeling, and false positive objects were considered less problematic than a missed metastasis.

For the segmentation task, the objective was to segment the WMHs on brain MRIs and get a reliable lesion volume estimation. The weights of the false positive and false negative pixels are set to the highest value (five), as the main goal was to reduce the incorrectly labeled pixels. The weights of the true positive pixels were set to 2, and the true negative pixels were set to 1, to handle class imbalance.

The range of values of the weights was based on the class weights during training of the base CNN, which were five and one for lesions and background, respectively.

### 3.6 Uncertainty of CNN

The uncertainty of the CNN in detecting or segmenting the lesions was assessed by the SD in probability outcome of the S layer. We implemented Monte Carlo dropout during test time,^24^^,^^25^ repeating the test phase 25 times. The SD of the probabilities over the 25 repetitions was calculated for every voxel. For the detection task, the mean SD of the detected metastases was calculated as an uncertainty metric to study the change in network uncertainty for detecting metastases. For the segmentation task, the maximum SD of all voxels in an image was taken as uncertainty metric to study the change in network uncertainty for the entire image.

## 4 Results

### 4.1 Liver Metastases Detection

#### 4.1.1 Number of iterations

In Table 1, the TPR, FPC, and the F1 score are reported for different numbers of iterations during the FT step. For 50 iterations, the TPR was similar to the TPR of the base CNN, but for 100 iterations and higher, the TPR improves. However, for more than 50 iterations, the FPC increased slightly. No significant differences (Wilcoxon signed-rank test with p<0.01) were found between the base CNN and the fine-tuned CNN for different numbers of iterations. Based on these results, the CNN was fine-tuned for 100 iterations for subsequent experiments.

**Table 1:** Median (IQR) of the TPR, FPC, and F1 score of the liver metastases detection for a varying number of iterations of learning for the CNN for FT. The best results are printed in bold.

	TPR	FPC	F1 score
Base CNN	0.67 [0.32 to 1.00]	3 [0 to 7]	0.49 [0.21 to 0.67]
50 iterations	0.67 [0.20 to 1.00]	**1 [0 to 3]**	0.40 [0.25 to 0.80]
100 iterations	0.85 [0.44 to 1.00]	2 [0 to 3]	**0.57 [0.40 to 0.67]**
500 iterations	**0.92 [0.33 to 1.00]**	2 [0 to 4]	0.50 [0.25 to 0.71]
1000 iterations	0.85 [0.50 to 1.00]	1 [0 to 4]	0.50 [0.40 to 0.75]

#### 4.1.2 Number of slices

The median (IQR) of the TPR, FPC, and the F1 score for analyses based on different numbers of slices are given in Table 2. The slice selection considered either all liver slices or all slices containing metastases. The slices with an average Softmax probability closest to 0.5 were selected.

**Table 2:** Median (IQR) of the TPR, FPC, and F1 score for a ranging number of slices presented to the CNN for FT. The best results are printed in bold. No significant differences were found between the Base CNN and all options.

	TPR	FPC	F1 score
Base CNN	0.67 [0.32 to 1.00]	3 [0 to 7]	0.49 [0.21 to 0.67]
1 slice liver	0.50 [0.11 to 1.00]	4 [1 to 8]	0.33 [0.12 to 0.64]
2 slices liver	0.42 [0.11 to 1.00]	1 [0 to 5]	0.33 [0.15 to 0.67]
1 slice metastases	0.67 [0.08 to 1.00]	4 [2 to 6]	0.31 [0.00 to 0.50]
2 slices metastases	0.67 [0.08 to 1.00]	3 [1 to 5]	0.31 [0.00 to 0.44]
All metastases slices	**0.85 [0.44 to 1.00]**	2 [0 to 3]	**0.57 [0.40 to 0.67]**
All liver slices	0.67 [0.20 to 1.00]	**0 [0 to 3]**	0.56 [0.33 to 0.80]

Including all the slices with liver metastases for CNN FT gave the best results for liver metastasis detection. FT the CNN with only one or two selected slices of the liver did not improve the results.

#### 4.1.3 Weighting

The median (IQR) of the TPR, FPC, and F1 score for the base CNN, fine-tuned CNN without weights, and fine-tuned CNN with weights are given in Table 3. The FT was done for 100 iterations and included all metastases slices. Putting more weight on the missed liver metastases led to more detected metastases, but at the cost of more false positive objects.

**Table 3:** Median (IQR) of the TPR, the FPC and the F1 score of the liver metastases detection, for weighting the true positives, false negatives, and false positives during the patient-specific FT. The best results are printed in bold.

	TPR	FPC	F1 score
Base CNN	0.67 [0.32 to 1.00]	3 [0 to 7]	0.49 [0.21 to 0.67]
FT CNN	0.85 [0.44 to 1.00]	**2 [0 to 3]**	**0.57 [0.40 to 0.67]**
Weighted FT CNN	**1.00 [0.62 to 1.00]**	6 [3 to 14]*	0.42 [0.29 to 0.57]

* Indicates a significant difference with results of the “base CNN” (Wilcoxon signed rank test with p<0.01).

#### 4.1.4 Qualitative results

**Fig. 3:**
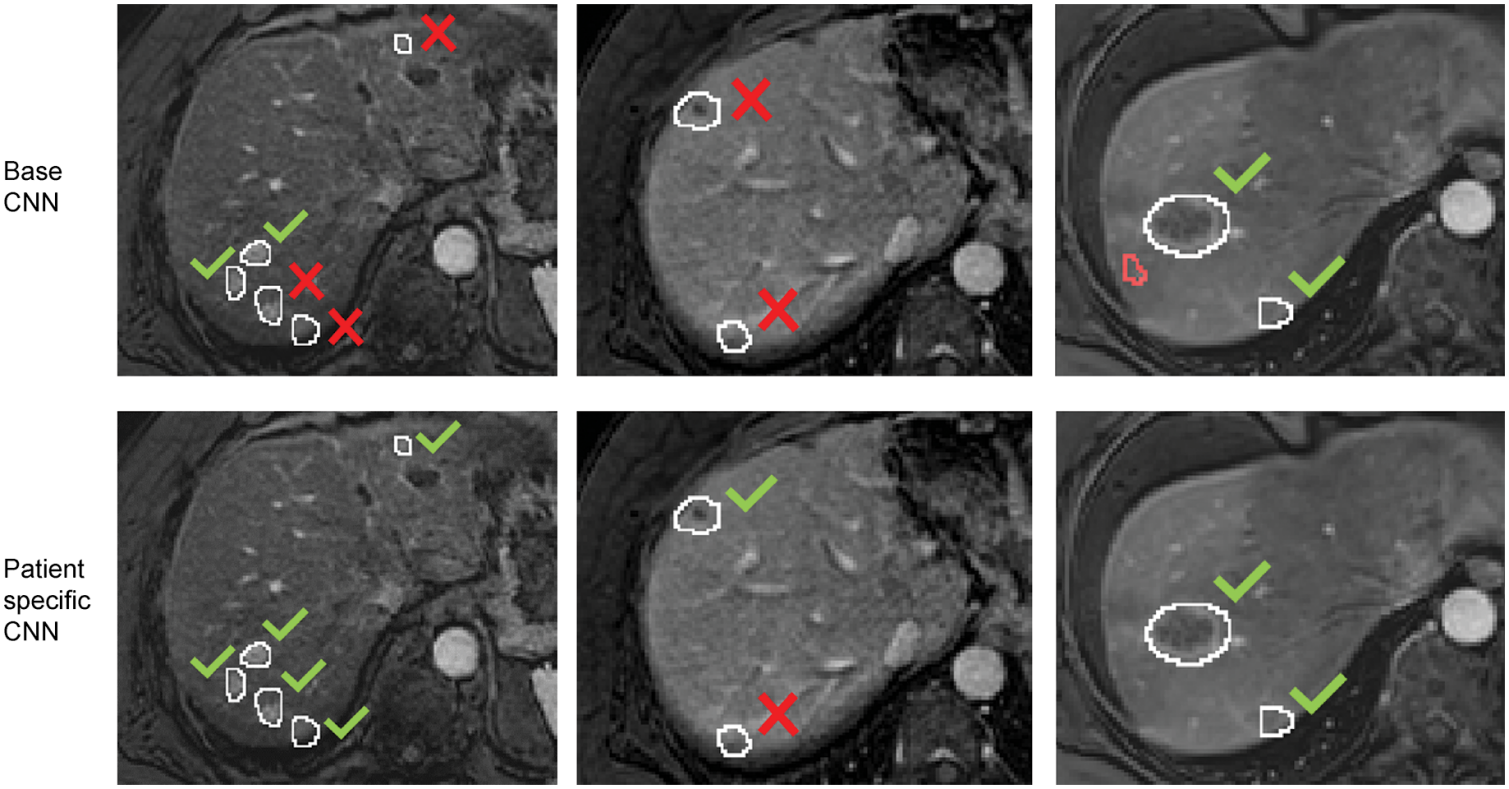
Examples of the detection results on the follow-up scan of the base CNN and the patient-specific CNN for three different patients. White outline = manual annotation, red outline = false positive object, green check = detected metastasis, red cross = missed metastasis.

Some visual examples of lesion detection by the base CNN and by the patient-specific CNN are shown in Fig. 3. The patient-specific CNN was fine-tuned in 100 iterations, including all slices with metastases present and without weights. After patient-specific FT the median TPR increased from 0.67 to 0.85, the median FPC decreased from 3 to 2, and the F1 score increased from 0.49 to 0.57.

Metastases smaller than $1\;cm^{3}$ were more often missed by the base CNN than larger metastases. An example is shown in the first column of Fig. 3. The TPR for the small metastases increased from 0.13 to 0.26 after patient-specific FT, while the TPR of the large metastases only increased slightly from 0.81 to 0.83. The increase in TPR was thus mainly due to the higher TPR of small metastases.

#### 4.1.5 Uncertainty of CNN

The Softmax probabilities of the detected metastases by the base CNN have a mean SD of 0.203 (±0.078). The mean SD can be considered a measure of the uncertainty of detection. The patient-specific CNN has a mean of 0.158 (±0.070), which is significantly lower than the mean SDs of the base CNN (p=0.003, paired Student’s t-test). The patient-specific CNN not only detects more metastases, it is also more certain about the detected metastases.

### 4.2 WMH Segmentation

#### 4.2.1 Number of iterations

All four options (50, 100, 500, or 1000 iterations) for the duration of the FT gave similar results. The Dice score significantly increased (paired Student’s t-test with p<0.01), and the AVD decreases after FT in comparison with the results of the base CNN. The base CNN had a mean DSC of 0.82 and a mean AVD of 20.7%. The fine-tuned CNNs had a mean DSC around 0.87 and a mean AVD around 10%. Using only 50 iterations gives a slightly lower Dice score (0.85) than the higher number of iterations. Based on these results, the number of iterations was set to 100 for the rest of the experiments.

#### 4.2.2 Number of slices

The mean (±SD) of the Dice score and the AVD for different numbers of slices are given in Table 4. The slice selection method selected the same slices when either all brain slices or all WMH slices were considered for selection. The selected slices originated mostly from slices covering the lateral ventricles and other regions with large WMH.

**Table 4:** Mean (±SD) of the Dice score and AVD of the WMH segmentation for a varying number of slices for FT. The best results are printed in bold.

	Dice score	AVD (%)
Base CNN	0.82±0.05	20.7±13.5
1 slice	0.86±0.04*	11.2±8.2
2 slices	0.86±0.04*	10.7±7.9
All lesion slices	0.87±0.04*	10.7±7.3
All brain slices	0.87±0.04*	10.2±6.9*

* Indicates a significant difference with results of the base CNN (paired Student’s t-test with p<0.01).

Including one slice already improved the Dice score and the AVD significantly, while adding more brain slices on top of the first one or two slices only gave a slightly better result. It is, therefore, possible to only annotate the one or two selected slices and improve the segmentation using these slices in the FT process.

#### 4.2.3 Weighting

The mean (±SD) of the Dice score and the AVD for the base CNN, fine-tuned CNN without weights, and fine-tuned CNN with weights are given in Table 5. Visual inspection of the results showed that weighting the true positive, false positive, and false negative pixels made the CNN more prone to label a pixel as a lesion, resulting in oversegmentation. For the WMH lesion segmentation task, weighting the pixels did not result in a better segmentation.

**Table 5:** Mean (±SD) of the Dice score and AVD of the WMH segmentation for weighting the true positives, false negatives, and false positives during the patient-specific FT. The best results are printed in bold.

	Dice score	AVD (%)
Base CNN	0.82±0.05	20.7±13.5
FT CNN	0.87±0.04*	10.7±7.3
Weighted FT CNN	0.86±0.05*	11.8±10.5

* Indicates a significant difference with results of the base CNN (paired Student’s t-test with p<0.01).

#### 4.2.4 Qualitative results

**Fig. 4:**
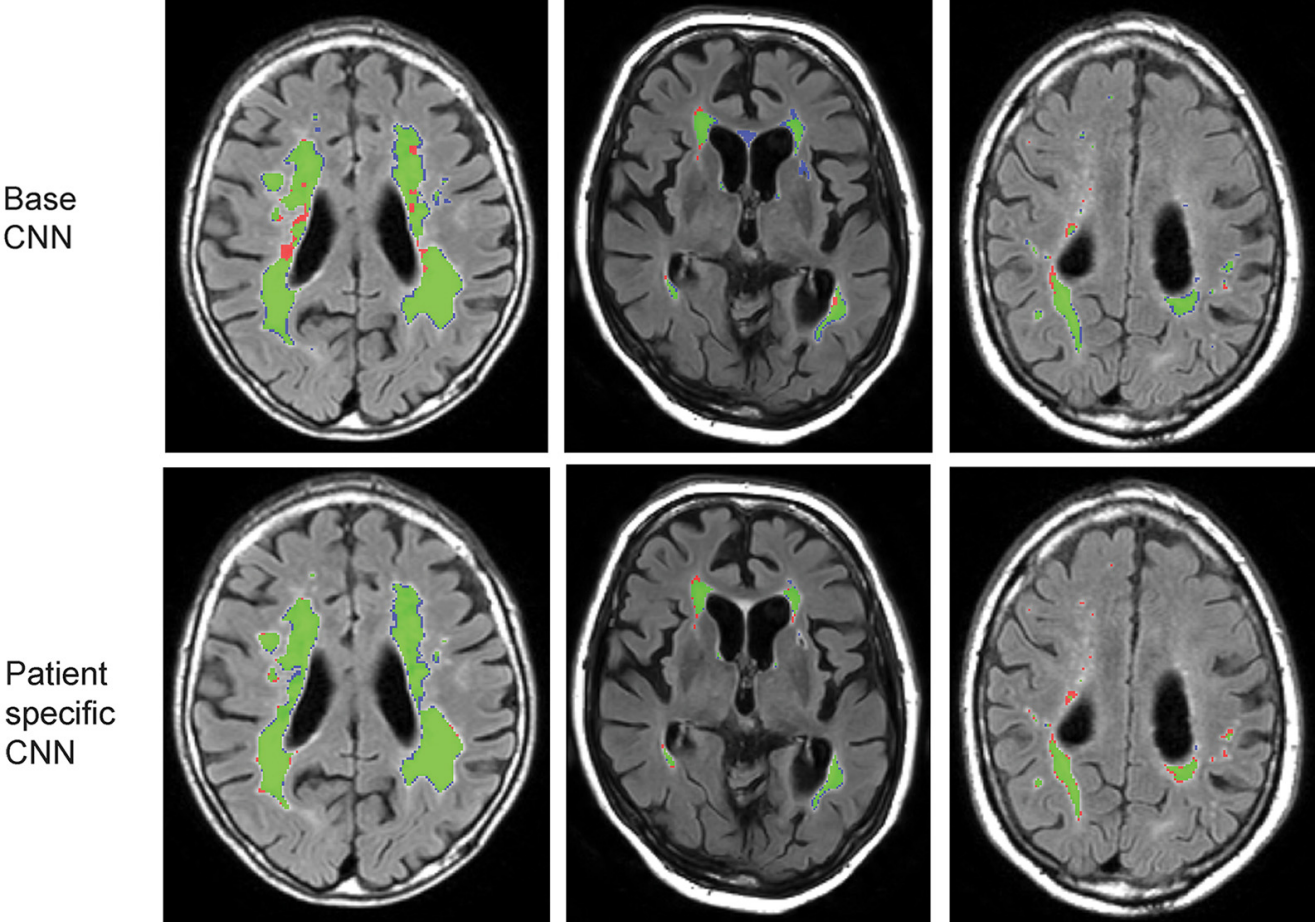
Examples of the follow-up scan with the segmentation results of the base CNN and the patient-specific CNN for three different patients. Green = true positive pixels, red = false negative pixels, and blue = false positive pixels.

Some visual examples of the lesion segmentation on the follow-up scan by the base CNN and the patient-specific CNN are shown in Fig. 4. The patient-specific CNN is fine-tuned using 100 iterations, including all slices with WMH lesions present and without weights. After patient-specific FT, the average Dice score increased from 0.82 to 0.87 and the AVD decreased from 20.7% to 10.7%.

Other metrics of evaluation in the WMH challenge are the modified Hausdorff distance, TPR, and F1 score.^20^ After patient-specific FT, the Hausdorff distance decreases from 2.40 to 2.01 mm, the TPR decreases from 0.84 to 0.63, and the F1 score remained 0.72. The TPR decreased because the fine-tuned CNN missed lesions with a size of only a few pixels. However, the other metrics improved due to the decrease in false positives and better volume segmentation.

The majority of the incorrectly labeled pixels by the base CNN were either small false positive regions (e.g., see Fig. 4, second column) or false negative pixels as part of larger lesions (e.g., see Fig. 4, first column). The patient-specific CNN learned to label these pixels correctly, resulting in a higher Dice score and lower AVD and thus providing a better WMH segmentation.

Smaller lesions were harder to correctly segment than larger lesions^20^ and we noticed that the patient-specific CNN missed more smaller lesions ($<0.01\;{\mathrm{cm}}^{3}$) than the base CNN in the segmentation, but at the same time also segmented fewer false positive pixels. The lesions and (noisy) false positive pixels labeled by the base CNN have a similar appearance, resulting in either labeling them all as lesions or all as background. The FT procedure seemed to put more emphasis on reducing the smallest false positives at the cost of small false negatives. The third column of Fig. 4 shows an example of these mislabeled pixels representing small lesions.

#### 4.2.5 Uncertainty of CNN

**Fig. 5:**
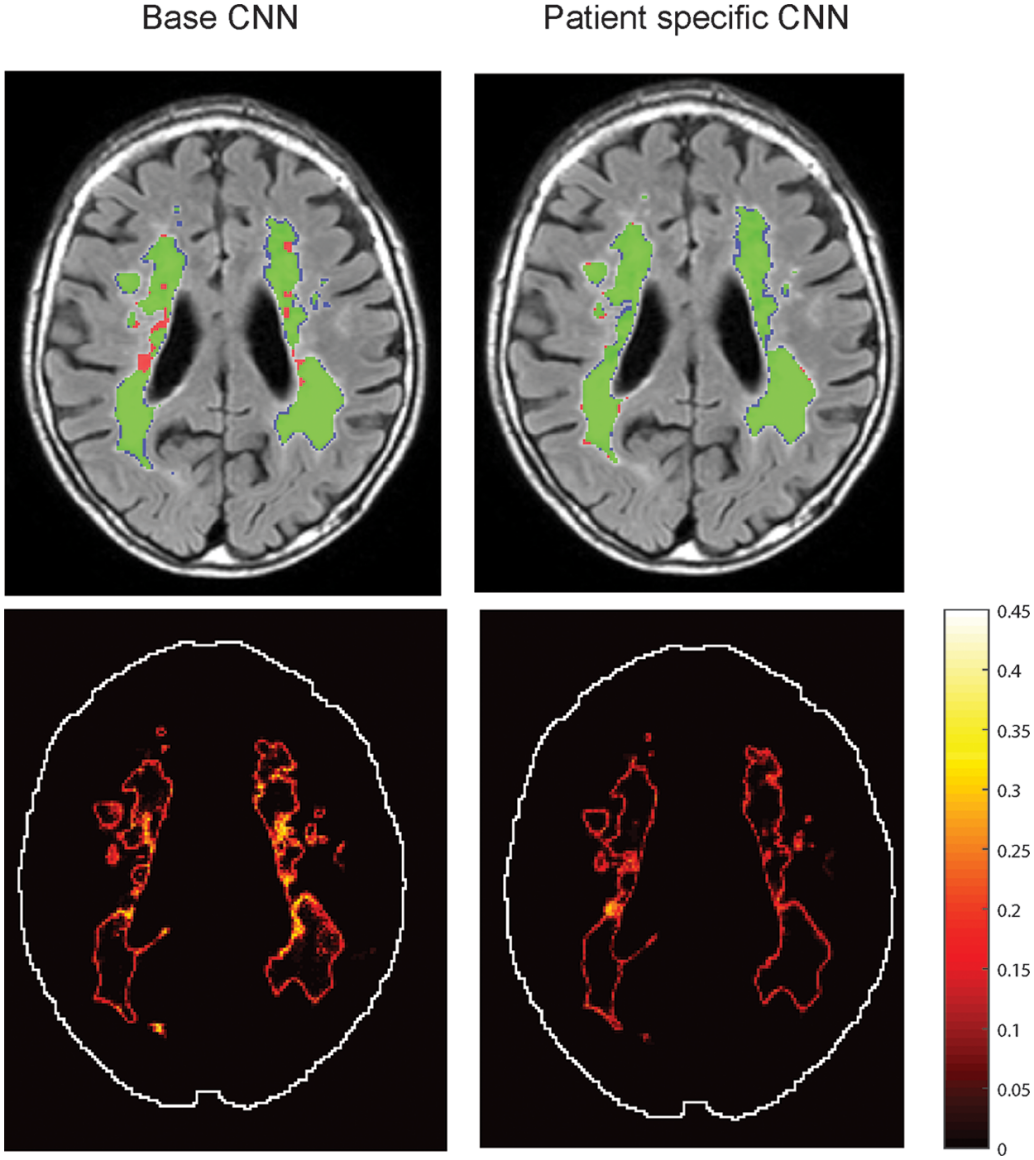
An example of the uncertainty (SD of Softmax probability) of the base CNN and the patient-specific CNN. A high SD means the CNN is uncertain about its decision.

The Softmax probabilities of the base CNN had a mean maximum SD of 0.398 (±0.025). The patient-specific CNN had a mean maximum SD of 0.328 (±0.038), which is significantly lower than the maximum SDs of the base CNN (p<0.001, paired Student’s t-test). The voxel with the maximum SD was always a lesion voxel. Both CNNs are thus more certain about nonlesion voxels than lesion voxels. In Fig. 5, an example is given of the mapped SD of the probabilities. The patient-specific CNN is more certain about the labels given, especially within large WMH.

## 5 Discussion

Patient-specific FT of a CNN based on previously acquired MRI yields improvements for quantification of lesions on subsequent MRI examinations. The TPR for the detection of liver metastases and the Dice score for the WMH segmentation both increased after patient-specific FT. Additionally, the certainty of the patient-specific CNN was higher than the certainty of the base CNN.

This study shows the potential of this patient-specific FT approach to improve lesion quantification results on follow-up imaging. Previously acquired scans of a patient seem to hold valuable information to refine a CNN accordingly, resulting in a better overall performance. The patient-specific FT step increased the lesion quantification effectiveness for two independent clinical applications (i.e., liver metastasis detection and brain WMH volume measurement), and we expect that this FT approach can also be applied in other clinical settings.

Some methodological considerations are important to note. The loss function calculated the errors on pixel level rather than object level. It was, therefore, more tailored toward the segmentation of liver metastases rather than the detection of the metastases. In case of large over- or undersegmentation on the baseline scan, it is most cost-effective for the CNN to correct for those areas. Large false positive regions are caused by tissue appearances that are not familiar to the base CNN, such as radiofrequency ablation regions in the liver resulting from earlier treatments. The patient-specific CNN will learn to correctly label the majority of the pixels in these regions. However, after patient-specific FT, pixels with a similar appearance but with a different underlying nature can be labeled incorrectly. This is an overshoot on the follow-up scan, where the patient-specific CNN corrects its results too much. False negatives on the baseline then might cause some false positives on the follow-up due to a similar appearance and vice versa. As a consequence, either more objects are detected incorrectly or more are missed. This might be overcome by tailoring the loss function toward the detection task, i.e., calculate the loss function on an object-level instead of on pixel level.

The patient-specific FT method assumes that the baseline and follow-up scan share features that describe the lesions and healthy tissue. However, there can be differences between the baseline scan and the follow-up scan due to (ongoing) treatment and variations in image acquisition. The patients from the brain WMH data set did not undergo any treatment that would be expected to influence WMH features, while most patients in the liver data set did. Therefore, the liver data set indeed shows more differences between the baseline and follow-up MRI scans than the brain data set. Variation in consecutive images increases the risk of overfitting on the baseline scan when the CNN is fine-tuned for many iterations. Fortunately, having more than 100 iterations for FT did not gain extra improvement compared to 100 iterations. The number of iterations should, therefore, be limited to reduce the risk of overfitting.

Patient-specific FT of the CNN may be prone to overfitting. However, in our experiments, no signs of overfitting were noticed, even when the baseline scan was visually very different from the follow-up scan. In such cases, the patient-specific CNN was still able to detect new lesions in the follow-up scan that were not initially detected by the base CNN. In addition, growing or shrinking lesions were also more reliably segmented in the follow-up scan.

Regarding the number of slices to include in the FT step, both applications (i.e., liver MRI and brain MRI) yielded the best results when all slices with lesions were included. However, for the WMH segmentation, the inclusion of only one or two slices gave similar results. WMH has similar features throughout the slices and the consecutive MRI exams. This resemblance makes it possible to fine-tune the CNN with only one or two slices, reducing the time and effort invested in annotating the slices. The liver metastases have different features throughout the slices and MRI exams, requiring to fine-tune the CNN with all metastases slices.

Inclusion of all slices of the organ is only beneficial if there is a balance between the lesion and nonlesion classes. An imbalanced data set arose for the liver metastasis detection when all the liver slices are included in the FT step. That is, on average, only 30% of the liver slices contain liver metastases, while 63% of the brain slices contain WMH. The CNN will then learn to distinguish different types of background (i.e., nonlesioned tissue) instead of the appearance of liver metastases, leading to fewer false positives and unfortunately also fewer true positives. In case of an imbalanced data set, a full lesion annotation is necessary to select the slices with lesions and subsequently gain improvement with patient-specific FT.

Moreover, the difference in the percentage of slices containing lesions leads to a considerable difference in the chance that a slice with lesions is selected. This resulted in the selection of slices without liver metastases and the unsuccessful FT of the CNN. Since brain MRI contains more slices with WMH, the chance to automatically select slices with WMH present is higher, resulting in a more successful FT of the CNN.

Weighting the true positives and false positives makes the detection CNN more inclined to label a pixel as a lesion. This results in more detected lesions, but also more false positives in comparison with the nonweighted patient-specific CNN. Considering that this approach is primarily aimed at lesion detection, one might accept more false positive objects. However, for the WMH segmentation, the weighting of the false positives, false negatives, and true positive pixels did not result in a better segmentation.

Information on lesion location found in the baseline scan could be of additional value for the detection method. In this study, we only considered the appearance of the lesion and nonlesion tissue. Adding the spatial information of former lesions might improve the detection rate and accuracy. Image registration could be used to allow for comparison between baseline and follow-up scans. However, such a method should also take into account that more lesions can develop or that lesions can disappear due to treatment.

This study shows the feasibility and power of patient-specific FT of a CNN. Experiments were done using a dual-pathway CNN in this study, but this approach can also be applied to other network architectures or loss functions.

## 6 Conclusion

In conclusion, patient-specific FT of a CNN for the quantification of lesions is a viable option to enhance the performance of the method using previously acquired data of the same patient. The CNN is fine-tuned toward that specific patient resulting in a better performance of the CNN. It is important that slices with lesions that represent the features of all lesions are included in the patient-specific FT step and to avoid imbalanced classes in these slices. In doing so, the patient-specific CNN detected more liver metastases than the base CNN, with the TPR increasing from 0.67 to 0.85. The patient-specific CNN also improved WMH segmentation with the Dice score increasing from 0.82 to 0.87.
